# Water-Soluble Cellulose Acetate Changes the Intestinal Microbiota in Mice with Non-Alcoholic Steatohepatitis

**DOI:** 10.3390/nu17030500

**Published:** 2025-01-29

**Authors:** Ayaka Iida, Ena Takahashi, Sachi Kuranuki, Shu Shimamoto, Tsuyoshi Nakamura, Hiroshi Kitagaki

**Affiliations:** 1 School of Nutrition and Dietetics, Faculty of Health and Social Services, Kanagawa University of Human Services, 1-10-1 Heisei-cho, Yokosuka 238-8522, Japan; 62312003.gc8@kuhs.ac.jp (E.T.); kuranuki-s@kuhs.ac.jp (S.K.); 2 Daicel Corporation, Konan 2-18-1, Minatoku, Tokyo 108-8230, Japan; sh_shimamoto@jp.daicel.com; 3 Department of Food and Health Sciences, International College of Arts and Sciences, Fukuoka Women’s University, 1-1-1, Kasumigaoka, Higashi-ku, Fukuoka 813-8529, Japan; t-naka@fwu.ac.jp; 4 Faculty of Agriculture, Saga University, Honjo-cho, 1, Saga 840-8502, Japan; ktgkhrs@cc.saga-u.ac.jp

**Keywords:** microbiota, non-alcoholic steatohepatitis, non-alcoholic fatty liver disease, water-soluble cellulose acetate

## Abstract

**Objectives**: Non-alcoholic fatty liver disease (NAFLD) is a prevalent chronic disorder of the liver and affects many people worldwide. Intestinal bacteria are thought to be involved in the pathological progression of NAFLD; therefore, improving the intestinal microbiota may be important in controlling NAFLD. In this study, we assessed the effects of water-soluble cellulose acetate (WSCA) on the intestinal microbiota in a non-alcoholic steatohepatitis (NASH) mouse model. **Methods**: NASH model (STAM mice) was created by streptozotocin injection and feeding the mice a high-fat diet. The serum biochemical parameters were analyzed. Intestinal bacterial populations were analyzed using paired-end sequencing of 16S rRNA, 18S rRNA, and internal transcribed spacer gene. **Results**: Our findings indicated that WSCA administration tends to improve the serum alanine aminotransferase and glucose levels in STAM mice and decreased the alpha diversity and altered the beta diversity of their intestinal microbiota. Additionally, WSCA intake resulted in an increase in the abundance of *Coriobacteriaceae_UCG-002* and a decrease in the abundance of *Enterobacter*. **Conclusions**: WSCA intake can alter specific microbial compositions to improve blood glucose levels and liver functions and may improve the pathogenesis of NAFLD.

## 1. Introduction

Non-alcoholic fatty liver disease (NAFLD) is a prevalent chronic liver condition in humans that has the potential to progress to non-alcoholic steatohepatitis (NASH), fibrosis, cirrhosis, and hepatocellular carcinoma (HCC) [1,2]. The incidence of NAFLD is increasing worldwide because the prevalence of obesity and type-2 diabetes is increasing [3,4]. The prevalence of NAFLD has been reported to be approximately 30%, based on global regional data collected from 1990 to 2019 [5]. As the prevalence of NAFLD rises, the number of cases of cirrhosis and HCC is expected to increase.

In 1980, Day et al. [6] proposed the “two-hit theory” as an onset mechanism of NASH, which states that obesity and insulin resistance lead to the accumulation of fat in the liver (first hit), and the addition of factors such as inflammatory cytokines and oxidative stress (second hit) cause progression to steatohepatitis. However, the “multiple parallel hits hypothesis” [7] proposed in 2010 has recently gained traction as an alternative explanation. This hypothesis states that multiple factors, including inflammatory cytokines, intestinal bacteria, dietary factors, and genetic factors, act concurrently on the liver, leading to the progression of fatty liver disease and NASH. Intestinal bacteria are involved in the pathological progression of NAFLD through endotoxins derived from intestinal bacteria and endogenous ethanol from ethanol-producing bacteria [7,8,9].

Balancing the intestinal microbiota and increasing the production of short-chain fatty acids are important for maintaining the intestinal environment [10]. Such effects are obtained by the ingestion of indigestible ingredients such as water-soluble dietary fiber. Water-soluble dietary fiber is more easily fermented and metabolized by intestinal bacteria than insoluble diet, and is considered to be useful for human health, such as reducing the risk of gastrointestinal diseases [11]. Epidemiology studies have reported that increased fiber intake may prevent NAFLD [12]. Thus, dietary fiber intake may improve the gut microbiota and inhibit the progression of NASH; however, the factors controlling the gut microbiota that may improve NASH are not yet fully understood.

Water-soluble cellulose acetate (WSCA; Daicel Corporation, Osaka, Japan) is a recently developed type of edible water-soluble dietary fiber, which comprises cellulose acetate made water-soluble by ester-bonding cellulose with acetic acid (degree of substitution: 0.6–0.9) [13]. We have previously reported that WSCA intake restores the intestinal microbiota to the normal range at the phylum level and improves serum ALT and glucose levels in a NASH mouse model [14]. However, the mechanism of these changes in intestinal microbiota have not been fully investigated. Therefore, in this study, we aimed to analyze the changes in WSCA intake on the intestinal microbiota of NASH model mice to determine which specific intestinal microbiota are affected.

## 2. Materials and Methods

### 2.1. Animal Experimentation

Fourteen-day-old pregnant female C57BL/6J mice were obtained from Charles River Japan (Yokohama, Japan). NAFLD model STAM mice were generated as previously described [15]. Briefly, NASH was induced in male mice by administering a single subcutaneous injection of 200 μg of streptozotocin (Sigma-Aldrich, St. Louis, MO, USA) 2 days postnatally. The mice were then fed High Fat Diet 32 ([HFD32]; CLEA Japan, Tokyo, Japan) ad libitum beginning at 4 weeks of age. STAM mice were randomly divided into four groups at 5 weeks of age (*n* = 5–6). One group was maintained as a control and only received HFD32 and the other three groups received food, with 3% of the cellulose in HFD32 replaced with WSCA, digestion resistant dextrin (DE), and fructo-oligosaccharide (OL). The diet composition is shown in Table 1. One or two animals were housed per cage.

The normal group consisted of male C57BL/6J mice that did not receive a streptozotocin injection and were administered AIN-93G (Oriental Yeast Co., Ltd., Tokyo, Japan) after weaning (*n* = 6). All mice were housed in a room maintained at 23 ± 2 °C on a 12 h light/dark cycle and were provided with ad libitum access to water. After fasting overnight, we dissected the mice at eight weeks (previously confirmed as the time at which STAM mice develop NASH [15]) under anesthesia by isoflurane inhalation (Wako Pure Chemical Industries, Ltd., Osaka, Japan). Blood and cecum tissue samples were collected. Blood was centrifuged at 12,000 rpm for 10 min at 4 °C and the serum was collected and stored at −30 °C. The cecum was rapidly frozen and stored at −80 °C.

This study was approved by the experimental animal ethics committee of Fukuoka Women’s University on 24 July 2018 (Approval Number: H30-4). All institutional and national guidelines for the care and use of laboratory animals were followed. The total number of mice in the experiment was 29.

### 2.2. Analysis of Blood Biochemistry

The serum levels of aspartate aminotransferase (AST), alanine aminotransferase (ALT), triglyceride (TG), total cholesterol (TCHO), and glucose (GLU) were assayed using FUJI DRI-CHEM7000 (Fujifilm Co., Tokyo, Japan) according to the manufacturer’s instructions.

### 2.3. Intestinal Bacteria Analysis

The intestinal microbiota of three individuals in each group were analyzed by Chemical Dojin Co., Ltd. (Kumamoto, Japan). Total genome DNA was extracted from samples using the CTAB/SDS method. DNA was diluted to 1 ng/μL using sterile water. 16S rRNA/18S rRNA/ITS genes of distinct regions were amplified using specific primers. PCR reactions were performed with Phusion^®^ High-Fidelity PCR Master Mix (New England Biolabs, Ipswich, MA, USA). The quantitative and qualitative determination of PCR products was performed by mixing the same volume of 1X loading buffer containing SYB green with PCR products and conducting electrophoresis on 2% agarose gel for detection. PCR products were mixed in equidensity ratios. Then, the PCR product mixture was purified using the Qiagen Gel Extraction Kit (Qiagen, Hilden, Germany). Libraries were generated with NEBNext^®^ Ultra™ DNA Library Prep Kit for Illumina, quantified via Qubit and Q-PCR, and analyzed using the Illumina platform. Paired-end reads were merged using FLASH (V 1.2.7; http://ccb.jhu.edu/software/FLASH/ (accessed on 5 December 2024)), a very fast and accurate analysis tool. Quality filtering on the raw tags was performed under specific filtering conditions to obtain high-quality clean tags according to the QIIME (V 1.7.0) quality-controlled process. The tags were compared with the reference database (Gold database) using the UCHIME algorithm to detect chimera sequences. Finally, the chimera sequences were removed to obtain the effective tags. Sequences analysis was performed using Uparse software (Uparse v 7.0.1001) with all effective tags. Sequences with ≥97% similarity were assigned to the same operational taxonomic unit (OTU). A representative sequence for each OTU was screened for further annotation. For each representative sequence, Mothur software was performed against the SSUrRNA database of the SILVA database for species annotation. OTU abundance information was normalized using a standard sequence number corresponding to the sample with the least sequences. Subsequent analyses of alpha and beta diversity used this output normalized data and were conducted using QIIME and R software (Version 2.15.3).

Cluster analysis was preceded by principal component analysis (PCA), which was performed to reduce the dimension of the original variables using the FactoMineR package and ggplot2 package in R software (Version 2.15.3). Principal Coordinate Analysis (PCoA) was displayed by WGCNA package, stat packages and ggplot2 package in R software (Version 2.15.3). Unweighted Pair-group Method with Arithmetic Means (UPGMA) Clustering was performed as a type of hierarchical clustering method to interpret the distance matrix using average linkage and was conducted by QIIME software (Version 1.7.0).

### 2.4. Statistical Analyses

Statistical analyses of serum biochemical parameters were conducted using IBM SPSS Statistics (version 28). Data were expressed as the mean ± standard error of the mean and analyzed using the Tukey–Kramer test. A *p*-value of <0.05 was considered statistically significant.

## 3. Results

### 3.1. Serum Biochemical Parameters

Serum biochemical parameters are shown in Table 2. Serum levels of AST, ALT, and GLU were higher in the STAM groups compared to in the normal group, but those in the WSCA group were the lowest among the STAM groups. Serum TCHO levels were significantly higher in OL and DE groups than in the normal group.

### 3.2. Intestinal Microbiota

#### 3.2.1. Alpha Diversity

**Figure 1:**
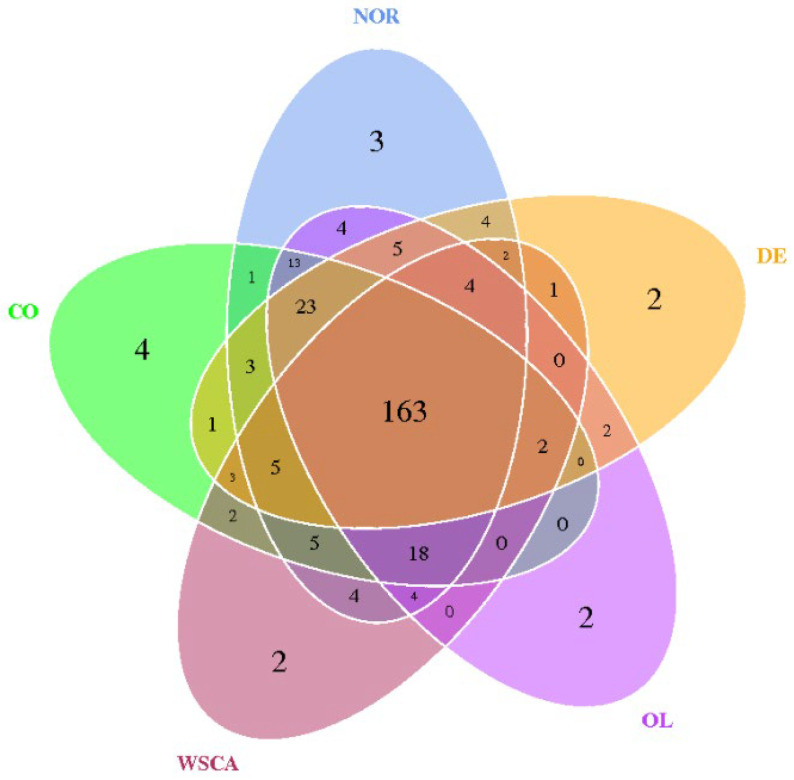
Venn diagram of operational taxonomic units (OTUs) in normal and STAM mice groups. NOR: normal group; CO: STAM and control group; WSCA: STAM and water-soluble cellulose acetate group; DE: STAM and digestion resistant dextrin group; and OL: STAM and fructo-oligosaccharide group.

**Figure 2:**
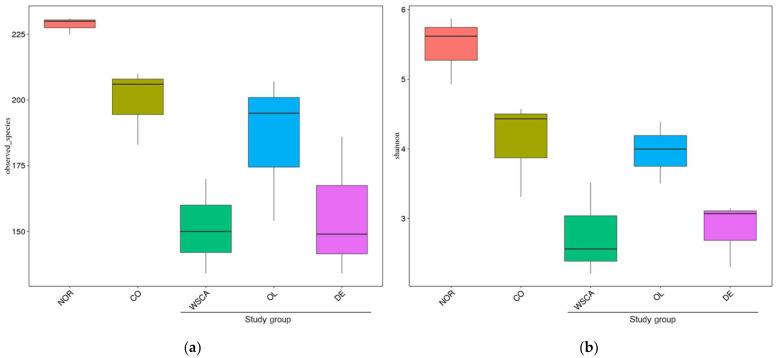
Difference in alpha diversity between normal and STAM mice groups based on (**a**) observed species and (**b**) Shannon indices. NOR: normal group; CO: STAM and control group; WSCA: STAM and water-soluble cellulose acetate group; DE: STAM and digestion resistant dextrin group; and OL: STAM and fructo-oligosaccharide group.

A Venn diagram based on the OTUs is shown in Figure 1. Each circle represents one group. All groups had 163 OTUs in common, with two to four OTUs unique to each group. Differences in the alpha diversity indices between groups are shown by boxplots based on observed species and Shannon indices (Figure 2). Alpha diversity was lower in the STAM groups than in the normal group, particularly in the WSCA and DE groups.

#### 3.2.2. Beta Diversity

**Figure 3:**
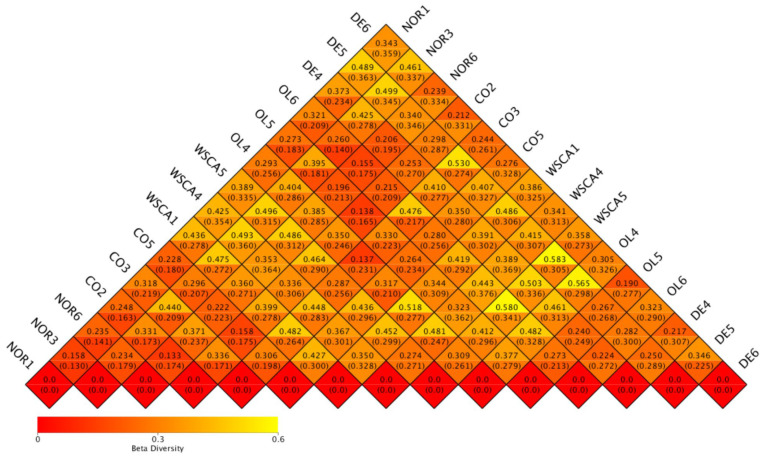
Beta diversity heatmap based on weighted UniFrac index (top of each square) and unweighted UniFrac index (bottom of each square). NOR: normal group; CO: STAM and control group; WSCA: STAM and water-soluble cellulose acetate group; DE: STAM and digestion resistant dextrin group; and OL: STAM and fructo-oligosaccharide group.

A heatmap based on the weighted and unweighted UniFrac indices is shown in Figure 3. Each grid represents the pairwise dissimilarity coefficient between pairwise samples, with the weighted Unifrac distance above and the unweighted Unifrac distance below. The weighted Unifrac distances were higher in the WSCA and DE groups than in the other groups.

**Figure 4:**
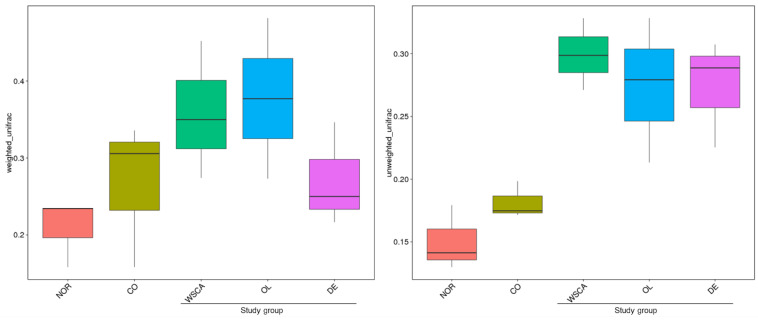
Boxplots showing the difference in beta diversity indices between normal and STAM mice groups based on weighted and unweighted UniFrac indices. NOR: normal group; CO: STAM and control group; WSCA: STAM and water-soluble cellulose acetate group; DE: STAM and digestion resistant dextrin group; and OL: STAM and fructo-oligosaccharide group.

The difference in beta diversity indices between groups is shown in Figure 4. In the boxplot based on the weighted UniFrac distance, beta diversity was higher in the WSCA and DE groups than in the normal and control groups. Conversely, in the boxplot based on the unweighted UniFrac distance, beta diversity was substantially higher in all the soluble fiber-substituted groups than in the normal and control groups.

**Figure 5:**
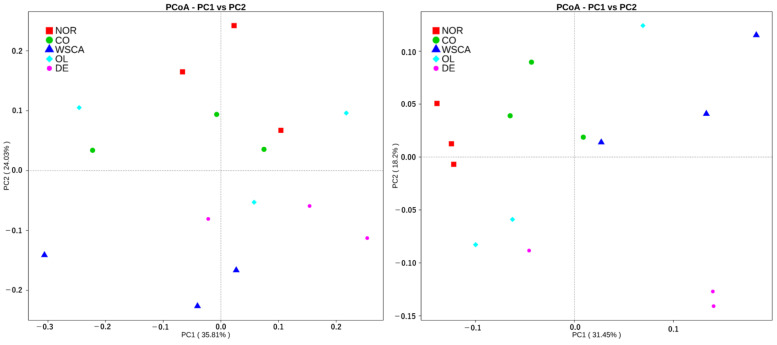
Principal coordinate analysis results showing the species composition similarity between normal and STAM mice groups based on (**Left**) weighted UniFrac and (**Right**) unweighted UniFrac indices. NOR: normal group; CO: STAM and control group; WSCA: STAM and water-soluble cellulose acetate group; DE: STAM and digestion resistant dextrin group; and OL: STAM and fructo-oligosaccharide group.

**Figure 6:**
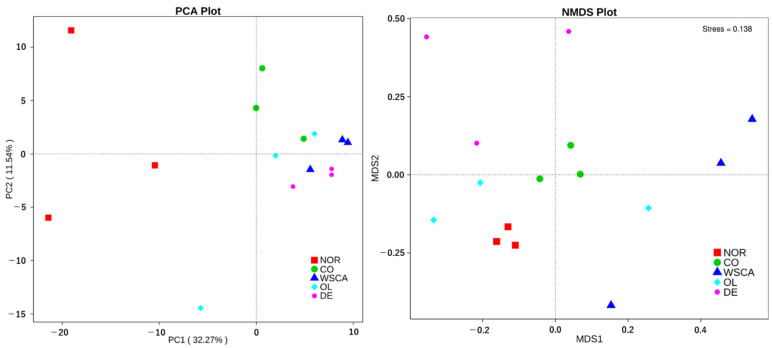
Principal component analysis and non-metric multi-dimensional scaling of normal and STAM mice groups. NOR: normal group; CO: STAM and control group; WSCA: STAM and water-soluble cellulose acetate group; DE: STAM and digestion resistant dextrin group; and OL: STAM and fructo-oligosaccharide group.

According to the PCoA results, combined samples have higher species composition similarity than individual samples. The WSCA and DE groups were dissimilar from the other groups and the OL group exhibited greater variability (Figure 5). According to PCA and non-metric multi-dimensional scaling (NMDS) results, the WSCA group was dissimilar from the normal group (Figure 6).

**Figure 7:**
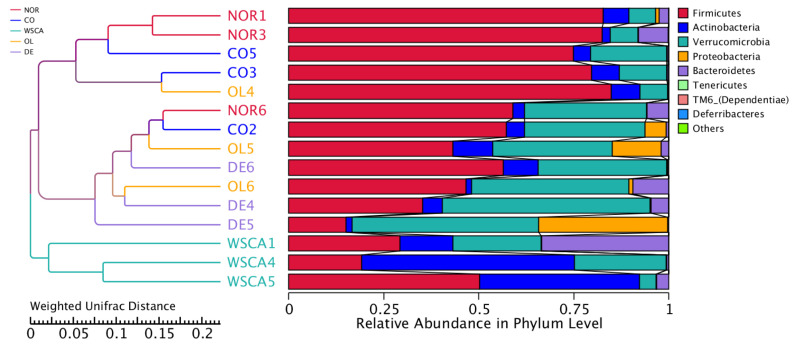

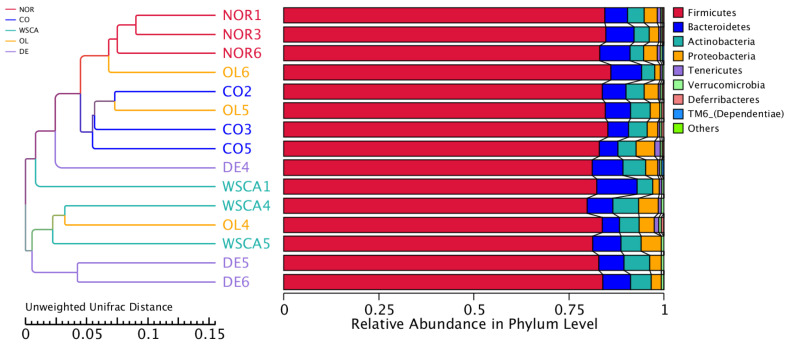
Unweighted pair-group method with arithmetic mean (UPGMA) cluster trees based on weighted and unweighted Unifrac distances. NOR: normal group; CO: STAM and control group; WSCA: STAM and water-soluble cellulose acetate group; DE: STAM and digestion resistant dextrin group; and OL: STAM and fructo-oligosaccharide group.

Unweighted pair-group method with arithmetic mean cluster trees based on weighted and unweighted Unifrac distances are shown in Figure 7. According to clustering based on the weighted UniFrac distance, the WSCA group was completely diverged from the other groups. Clustering based on the unweighted UniFrac distance also showed that the WSCA group diverged from both the normal and control groups.

#### 3.2.3. Analysis of Microbiota Composition at the Phylum Level

**Figure 8:**
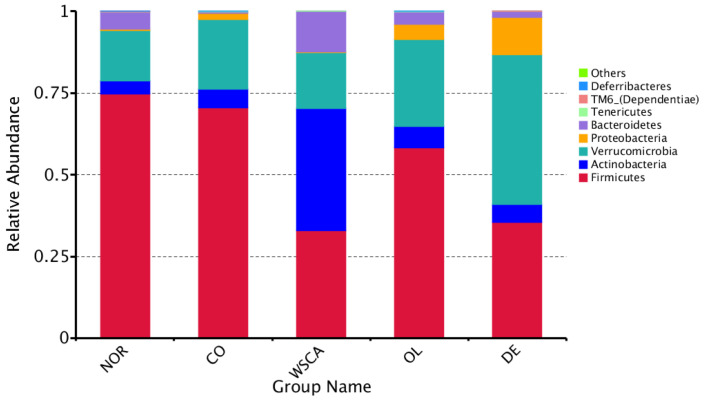
Relative abundance of species at the phylum level. NOR: normal group; CO: STAM and control group; WSCA: STAM and water-soluble cellulose acetate group; DE: STAM and digestion resistant dextrin group; and OL: STAM and fructo-oligosaccharide group.

Species relative abundance at the phylum level is shown in Figure 8. The abundance of Proteobacteria was higher in the control group compared to the normal group. Among the STAM groups, only the WSCA group did not exhibit an increased proportion of *Proteobacteria*. The abundance of *Actinobacteria* was higher in the WSCA group than in the other groups. The abundance of *Verrucomicrobiota* was higher in the DE group than in the other groups.

#### 3.2.4. Analysis of Microbiota Composition at the Genus Level

**Figure 9:**
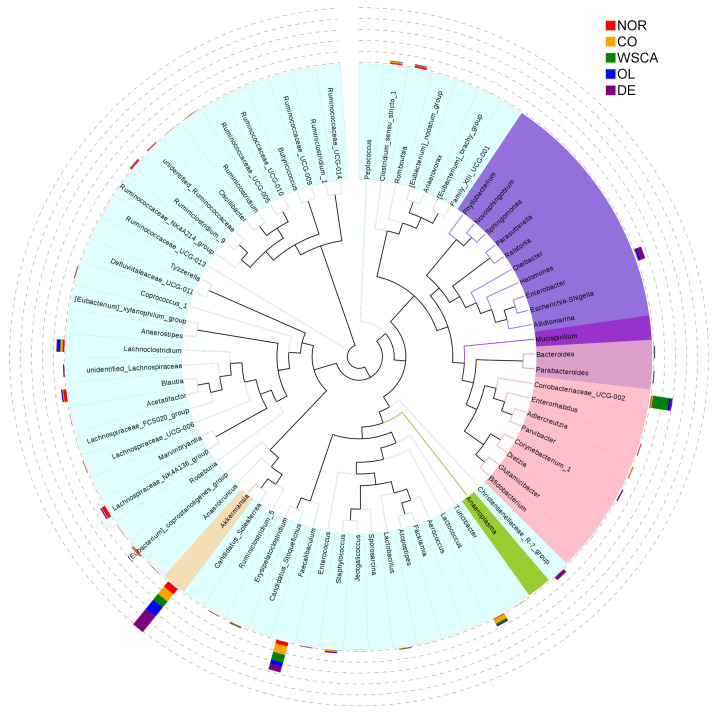
Evolutionary tree showing the taxonomic abundance at the genus level. NOR: normal group; CO: STAM and control group; WSCA: STAM and water-soluble cellulose acetate group; DE: STAM and digestion resistant dextrin group; and OL: STAM and fructo-oligosaccharide group.

**Figure 10:**
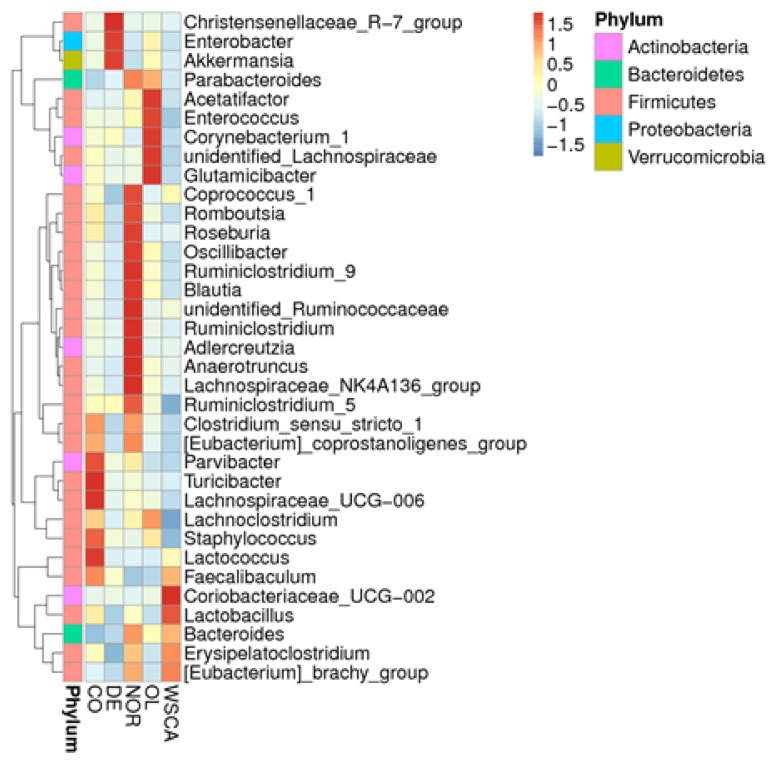
Heatmap showing the taxonomic abundance at the genus level. NOR: normal group; CO: STAM and control group; WSCA: STAM and water-soluble cellulose acetate group; DE: STAM and digestion resistant dextrin group; and OL: STAM and fructo-oligosaccharide group.

The evolutionary tree and heatmap at the genus level are shown in Figure 9 and Figure 10. In particular, the genera that were most abundant only in certain groups were *Akkermansia* (DE group), *Coriobacteriaceae_UCG-002* (WSCA group), *Lactococcus* (control group), *Enterobacter* (DE group), *Blautia* (normal group), and *Christensenellaceae_R-7_group* (DE group).

## 4. Discussion

The mechanisms of NASH pathogenesis are not fully understood; however, alteration in the intestinal microbiota is considered a cause of NAFLD pathological progression [7,8,9]. Therefore, we conducted this study using a mouse model of NASH to determine the effect of WSCA on gut microbiota as a strategy to treat and prevent NAFLD by targeting the gut microbiota. This study differs from our previous one by the dose of WSCA (1% or 2%) [14]. In addition, changes in intestinal microbiota, including other soluble fibers, were analyzed in detail, and then compared.

The results of liver function tests on AST and ALT serum levels showed that these biochemical indicators were elevated in the NASH groups than the normal group, but only the WSCA group showed an improvement. Similar results were obtained for serum GLU levels. The trend in serum AST levels was slightly different from our previous study, showing an improvement trend in the WSCA group [14]. However, the difference in dose may have affected the results, since the dose of WSCA in the previous study was 1%, and that of the current study is 3%. NASH is considered the hepatic phenotype of metabolic syndrome and is often accompanied by diabetes because it is associated with abnormal glucose metabolism [16,17]. STAM mice, i.e., the NASH model animal used in this study, also exhibit hyperglycemia [15]. Feeding STAM mice with a diet containing 3% WSCA for three weeks led to an improvement in serum GLU levels. Additionally, the ingestion of fructo-oligosaccharides and indigestible dextrin, which were used as a control for water-soluble dietary fiber ingredients currently permitted for specified health use in Japan, had no effect on liver function or blood glucose levels. In other words, these water-soluble dietary fibers showed minimal improvement in NASH pathology. Furthermore, no significant difference was observed between mouse groups regarding serum TG levels, which is an indicator of lipid metabolism, and WSCA intake also had no effect on TCHO levels. Although not related to water-soluble dietary fiber, a previous study reported that probiotics in the treatment of metabolic-related fatty liver disease in humans modulate carbohydrate metabolism rather than lipid metabolism and improve liver enzyme levels [18]. Similarly, in our study, changes in the intestinal microbiota caused by water-soluble dietary fiber intake did not influence lipid metabolism.

Alpha diversity is commonly employed to evaluate microbial diversity within a community [19,20]. In general, a host is considered healthy when there is high species diversity with a good balance of many bacteria types among intestinal microbiota [21]; however, various diseases such as inflammatory bowel disease and metabolic syndrome reduces alpha diversity [22]. Alpha diversity is also reduced when specific components act on intestinal bacteria. Alpha diversity has been reported to either increase or decrease due to increased dietary fiber intake [23]. In this study, the WSCA and DE groups exhibited a prominent decrease in alpha diversity among the NASH groups, which suggests that WSCA and indigestible dextrin affect specific intestinal microbiota. Additionally, almost no change was observed in the OL group compared to the control group, indicating that the intestinal microbiota was not significantly affected.

Beta diversity indicates the degree of differences in diversity between samples. In this study, beta diversity was shown using heatmaps, boxplots of UniFrac distances, PCoA, PCA, and NMDS based on the UniFrac distances. The heatmaps showed that the WSCA and DE groups did not resemble the intestinal microbiota of the other groups, indicating that it comprised unique microbiota. This was confirmed by the PCoA, PCA, NMDS, and clustering results. The WSCA and DE groups showed similar results for beta diversity as those for alpha diversity. However, a comparison of these groups using PCoA and clustering based on the weighted UniFrac distance showed that the WSCA group had more unique beta diversity. In other words, WSCA intake had a unique effect on the intestinal microbiota that was not induced by other water-soluble dietary fibers. Numerous animal studies have demonstrated that high-fat diets alter the beta diversity of intestinal bacteria [24,25,26]. Although the method of creation is different from the STAM mice used in this study, changes in β-diversity have also been reported in previous studies combining streptozotocin inoculation and a high-fat diet [27]. This study showed changes in beta diversity in the control group compared to the normal group, but each of the three soluble fiber intake groups showed specific changes in beta diversity.

We compared the intestinal microbiota at the phylum and genus levels. Compared with the normal group, the control group had a higher relative abundance of Proteobacteria. This result was consistent with that seen in human NASH [9]. Our genus-level heatmap results showed that only *Enterobacter* in the phylum Proteobacteria changed; thus, changes in the relative abundance of Proteobacteria at the phylum level are thought to reflect changes in *Enterobacter* at the genus level. *Enterobacter* are Gram-negative normal intestinal flora. Endotoxins, which are a constituent of the cell wall of Gram-negative bacteria, are released in large amounts when the cell wall breaks down. Actually, endotoxin levels have been reported to be elevated in patients with NAFLD [28]. The results of our study showed that the abundance of Proteobacteria increased in the control group compared to the normal group, suggesting that an increase in *Enterobacter* may be involved in the onset of NASH. Thus, approaches to reduce the abundance of *Enterobacter* will be needed in the future to control the development of NASH.

Of the NASH groups, only the WSCA group did not increase the relative abundance of Proteobacteria (i.e., *Enterobacter*) and it was maintained at the same level as that in the normal group. Additionally, the WSCA group was characterized by a higher abundance of Actinobacteria than the other groups. Genus-level heatmaps showed that the abundance of *Coriobacteriaceae_UCG-002* increased in the WSCA group. *Coriobacteriaceae_UCG-002* is thought to be involved in the production of acetic acid, a short-chain fatty acid [29], and regulates the intestinal environment. Both preclinical and clinical studies have reported that short-chain fatty acids stimulate the production of glucagon-like peptide (GLP-1), which is secreted from L cells in the small intestine [30,31]. GLP-1 reduces blood glucose levels by promoting insulin secretion and suppressing the production of glucagon, which leads to an increase in blood glucose levels [32,33]. However, the STAM mice used in this study constituted a pathology model characterized by the destruction of pancreatic β cells following streptozotocin inoculation, and it is unlikely that GLP-1 influenced insulin secretion [14,15]. In contrast, it has been reported that WSCA intake, albeit in rat experiments, increased acetate and propionate concentrations in a dose-dependent manner due to bacterial fermentation [34]. It is thought that the cellulose liberated from WSCA is degraded and fermented by bacteria as in the case of native cellulose in plant cell walls [34]. Therefore, we suggest that WSCA intake enhanced GLP-1 production by short-chain fatty acid production through an increase in the abundance of *Coriobacteriaceae_UCG-002* and/or the fermentation of WSCA by bacteria, which suppressed glucagon production and may have contributed to an improvement in hyperglycemia. Since impaired glucose tolerance is a disease state that underlies NAFLD, improving hyperglycemia is considered to subsequently lead to an improvement in liver function associated with NAFLD. Although short-chain fatty acids were not measured in this experiment, their potential role in this mechanism cannot be excluded.

The normal group was also characterized by a high abundance of *Blautia*. *Blautia* species are decreased in patients with diabetes and cirrhosis but increased in individuals with a smaller volume of visceral fat, producing butyric acid and acetic acid among short-chain fatty acids [35]. However, an increase in *Blautia* species was not observed in the WSCA group. Therefore, these results indicate that WSCA intake improved NASH pathology through characteristic microbiota rather than by improving the normal intestinal microbiota, which is similar to the beta diversity results.

Regarding the other water-soluble dietary fibers, the DE group, which showed similar changes in alpha and beta diversity to the WSCA group, was characterized by higher abundances of *Akkermansia* and *Christensenellaceae_R-7_group*, which are beneficial bacteria, compared to the other STAM groups. *Akkermansia* may be effective against obesity and type-2 diabetes [36,37,38], whereas *Christensenella* is attracting increasing attention because of its potential to suppress obesity [38,39]. In Japan, DE is approved as a food ingredient for specific health requirements as it moderates glucose absorption. In this study, although DE intake did not contribute to an improvement in NASH pathology, it may produce unique intestinal microbiota. In humans, the predominant genera within the *Bacteroidota* phylum are *Bacteroides* and *Prevotella*. *Prevotella* is involved in the degradation of dietary fiber and the production of short-chain fatty acids [40]. However, in this study, *Prevotella* was not identified as a characteristic member of the microbiota. Therefore, further research is required in the future.

Our findings indicate that soluble dietary fiber induces specific changes in the beta diversity of the intestinal microbiota in mice with NASH. While the results of this study are based on a NASH mouse model, they may have implications for human NAFLD as well. The observed changes in the intestinal microbiota suggest that interventions, such as diets enriched with specific dietary fibers targeting the gut microbiota, could be beneficial for human NAFLD. Furthermore, among soluble dietary fibers, WSCA intake may improve the pathogenesis of NASH; a WSCA-based approach to the gut microbiota may be a preventive or therapeutic method for NASH.

The limitation of this study is that the results were obtained only from STAM mice. The results may differ in other NAFLD models, such as genetically modified mice. Furthermore, as this study focused on changes in the intestinal microbiota, blood parameters were kept to a minimum to reflect the state of the disease. In the future, it will be necessary to conduct evaluations in combination with liver histology and other diagnostic tests. The long-term effects of WSCA have yet to be investigated; such investigations will take place in future. WSCA may have potential applications as a dietary fiber source for supplements and functional foods in the fields of food and nutrition.

## 5. Conclusions

WSCA intake in STAM mice, as a model of NASH pathogenesis, increased the relative abundance of *Coriobacteriaceae_UCG-002* and decreased the relative abundance of *Enterobacter*, and contributed to improved liver function and blood glucose levels. The effect of WSCA intake on NASH intestinal microbiota was unique relative to that observed from the intake of other water-soluble dietary fibers, indigestible dextrin, and fructo-oligosaccharides.

## Figures and Tables

**Table 1:** Components of the experimental diet (%).

Components	STAM (High Fat Diet [HFD32])			
	Control	WSCA	DE	OL
Milk casein	24.5	24.5	24.5	24.5
Powdered albumen	5.0	5.0	5.0	5.0
L-cystine	0.43	0.43	0.43	0.43
Powdered beef tallow (containing 80% beef tallow)	15.88	15.88	15.88	15.88
Safflower oil (high-oleic type)	20.0	20.0	20.0	20.0
Cellulose	5.5	2.5	2.5	2.5
Water-soluble cellulose acetate	-	3.0	-	-
Indigestible dextrin	-	-	3.0	-
Fructo-oligosaccharide	-	-	-	3.0
Maltodextrin	8.25	8.25	8.25	8.25
Lactose	6.928	6.928	6.928	6.928
Sucrose	6.75	6.75	6.75	6.75
AIN-93G mineral mixture	5.0	5.0	5.0	5.0
AIN-93 vitamin mixture	1.4	1.4	1.4	1.4
Choline hydrogen tartrate	0.36	0.36	0.36	0.36
t-Butylhydroquinone	0.002	0.002	0.002	0.002
Total	100.000	100.000	100.000	100.000

Control and only received HFD32 and the other three groups received food, with 3% of the cellulose in HFD32 replaced with water-soluble cellulose acetate (WSCA), digestion resistant dextrin (DE), and fructo-oligosaccharide (OL).

**Table 2:** Comparison of AST, ALT, TG, TCHO, and GLU serum levels in normal and STAM mice.

	Normal			STAM											
				Control			WSCA			DE			OL		
Number of Mice	6			6 *			5			6			6		
AST (U/L)	73.2	±	22.0 ^a^	303.2	±	51.5 ^b^	209.6	±	24.4 ^ab^	290.2	±	58.6 ^ab^	280.0	±	67.4 ^ab^
ALT (U/L)	34.2	±	11.9 ^a^	123.7	±	17.1 ^b^	84.2	±	10.7 ^ab^	126.2	±	21.5 ^b^	123.3	±	25.0 ^b^
TG (mg/dL)	120.7	±	13.3	197.4	±	99.2	263.6	±	99.7	220.8	±	54.3	348.7	±	81.4
TCHO (mg/dL)	105.2	±	9.8 ^a^	157.5	±	11.3 ^ab^	159.6	±	31.4 ^ab^	201.0	±	12.9 ^bc^	240.2	±	14.8 ^c^
GLU (mg/dL)	158.7	±	23.7 ^a^	599.0	±	53.3 ^bc^	444.6	±	86.0 ^b^	662.7	±	61.1 ^bc^	710.0	±	39.3 ^c^

Values are expressed as the mean ± standard error of the mean (SEM). Different lowercase letters indicate significant differences (*p* < 0.05). AST, aspartate aminotransferase; ALT, alanine aminotransferase; TCHO, total cholesterol; TG, triglyceride; GLU, glucose; WSCA, water-soluble cellulose acetate; DE, digestion resistant dextrin; OL, fructo-oligosaccharide * TG: *n* = 5.

## Data Availability

Data can be accessed from the corresponding author upon request.
